# Extracellular Adenine Nucleotides and Adenosine Modulate the Growth and Survival of THP-1 Leukemia Cells

**DOI:** 10.3390/ijms21124425

**Published:** 2020-06-22

**Authors:** Kamila Puchałowicz, Maciej Tarnowski, Marta Tkacz, Dariusz Chlubek, Patrycja Kłos, Violetta Dziedziejko

**Affiliations:** 1 Department of Biochemistry, Pomeranian Medical University in Szczecin, Powstańców Wlkp. 72 av., 70-111 Szczecin, Poland; dchlubek@pum.edu.pl (D.C.); patison@pum.edu.pl (P.K.); viola@pum.edu.pl (V.D.); 2 Department of Physiology, Pomeranian Medical University in Szczecin, Powstańców Wlkp. 72 av., 70-111 Szczecin, Poland; maciejt@pum.edu.pl (M.T.); tkacz.mag@gmail.com (M.T.)

**Keywords:** acute myeloid leukemia, apoptosis, cell cycle, chemoresistance, cytotoxicity, extracellular nucleotides, proliferation, purinergic signaling, SDF-1

## Abstract

A new approach to improve the effectiveness of acute myeloid leukemia (AML) treatment is to use the properties of purinergic signaling molecules secreted into the bone marrow milieu in response to leukemic cell growth. Therefore, our study aimed to evaluate the effects of extracellular adenine nucleotides and adenosine on the growth and death parameters in the leukemic THP-1 cell line. Cells were exposed to ATP, ADP, AMP, adenosine and nonhydrolyzable analogues of ATP and ADP (ATPγS and ADPβS) in a 1–1000 μM broad concentration range. The basal mRNA expression of the P1 and P2 receptors was evaluated by real-time PCR. Changes in the processes of cell growth and death were assessed by flow cytometry analysis of proliferation, cell cycle and apoptosis. Chemotaxis toward stromal cell-derived factor-1 (SDF-1) was performed using the modified Boyden chamber assay, and chemokine receptor type 4 (CXCR4) surface expression was quantified by flow cytometry. We indicated several antileukemic actions. High micromolar concentrations (100–1000 μM) of extracellular adenine nucleotides and adenosine inhibit the growth of cells by arresting the cell cycle and/or inducing apoptosis. ATP is characterized by the highest potency and widest range of effects, and is responsible for the cell cycle arrest and the apoptosis induction. Compared to ATP, the effect of ADP is slightly weaker. Adenosine mostly has a cytotoxic effect, with the induction of apoptosis. The last studied nucleotide, AMP, demonstrated only a weak cytotoxic effect without affecting the cell cycle. In addition, cell migration towards SDF-1 was inhibited by low micromolar concentrations (10 μM). One of the reasons for this action of ATPγS and adenosine was a reduction in CXCR4 surface expression, but this only partially explains the mechanism of antimigratory action. In summary, extracellular adenine nucleotides and adenosine inhibit THP-1 cell growth, cause death of cells and modulate the functioning of the SDF-1/CXCR4 axis. Thus, they negatively affect the processes that are responsible for the progression of AML and the difficulties in AML treatment.

## 1. Introduction

Acute myeloid leukemia (AML) is a heterogeneous group of hematological malignancies characterized by clonal proliferation and the accumulation of morphologically and functionally immature myeloid progenitor cells in bone marrow and peripheral blood [1]. It is the most common leukemia in adults; the incidence increases significantly after 45 years of age [2,3]. In developed countries, each year, 4–8 new cases are diagnosed per 100,000 inhabitants, and diagnoses occur slightly more often in men than in women [4,5]. Although there has been a continuous increase in the survival rate of patients with AML in the last four decades, they have the lowest survival rates of all types of leukemia, which decreases significantly with age [1,3]. From 2009–2015, the estimated 5-year survival rate in the U.S. was 28.3% [3]. The low estimated 5-year survival rate is related to AML’s aggressive course, the high risk of relapse and the insufficient effectiveness of the currently available treatment regimens. An important limitation of treatment, especially in older patients, is the high toxicity of the cytostatics used [1]. Difficulties associated with AML treatment result from the etiopathogenesis of this heterogeneous group of cancers. The accumulation of acquired genetic disorders and epigenetic changes in hematopoietic cells leads to signal transduction and gene expression defects, and, consequently, to abnormalities in key hematopoiesis processes, such as self-renewal, proliferation, differentiation and apoptosis [6]. Furthermore, the interaction between stromal cell-derived factor-1 (SDF-1 or CXCL12) and chemokine receptor type 4 (CXCR4) is crucial for the migration and adhesion of leukemia cells to the bone marrow stroma. The accumulation of leukemic cells in bone marrow and the decrease in their sensitivity to drugs is the main cause of minimal residual disease (MRD) and an increased risk of recurrence [7]. Despite numerous studies devoted to the development of new AML therapies, treatment methods have not changed significantly for several decades. Therefore, it is necessary to further deepen our knowledge about the complex biology of AML cells and to search for new drug targets that could help to improve long-term outcomes in AML patients.

Other properties besides the properties of cancer cells also have a significant impact on the course of cancer. Another important factor is the biochemical and cellular composition of the bone marrow microenvironment, whose role is increasingly emphasized in the case of hematological malignancies [8,9]. Purine nucleotides and nucleosides are significant components of the bone marrow microenvironment of AML [10]. Adenine nucleotides are released into the extracellular space from necrotic and inflammatory cells or cancer cells [11]. Their concentration in healthy tissues is very low (nanomolar range), whereas their concentration increases in the cancer microenvironment to the micromolar range (hundreds) [12]. Over the course of neoplastic growth, adenine nucleotides and adenosine (1) are abundant components of the tumor microenvironment; (2) are potent modulators of the immune response and cytokine release (alarmins); (3) play a key role in host–cancer interactions; and (4) modulate the growth of cancer cells [13].

Extracellular adenine nucleotides and their breakdown product, adenosine, can trigger many different cell responses, including cell adhesion, migration, proliferation, differentiation and death in normal cells [10,14,15,16] and leukemic bone marrow cells [15,17]. ATP and ADP act by binding to the P2 receptor family, which is subdivided into two subgroups: P2X, a ligand-gated ion channel receptor (only for ATP), and P2Y, a G protein-coupled receptor (for ATP, ADP, UTP, UDP or UDP–glucose) [18]. Adenosine mediates effects mainly through interaction with the G protein-coupled P1 receptor family [19]. AML cells are characterized by altered expression of nucleotide receptors [20,21,22] and CD39 (ecto-nucleoside triphosphate diphosphohydrolase 1 [E-NTPDase1]) and CD73 (ecto-5′-nucleotidase) ectonucleotidases [23], compared to normal cells. This is a mechanism that protects leukemia cells from elimination via purinergic signaling. Interestingly, AML cells (blasts and leukemic stem cells (LSCs)) respond differently to high ATP concentrations (1–5 mM) compared to hematopoietic stem cells (HSCs) [17,22,24]. ATP induces cell cycle arrest, inhibits proliferation [22], and induces apoptosis in AML cells [17,22], but not in HSCs. Any differences in the properties of AML cells compared to normal cells are potential new drug targets.

Therefore, in our study, we present the effects of extracellular adenine nucleotides and adenosine on the parameters of cell growth and death in the well-characterized AML cell line, THP-1. THP-1 is a human acute monocytic leukemia cell line that was originally established from the peripheral blood of a 1-year-old male. It is a valuable experimental model for the investigation of cell response to potential antileukemic compounds and the identification of new drug targets in studies on AML. We focused on the cell features that are associated with significant clinical problems, such as rapid progression of AML and chemoresistance. These include uncontrolled proliferation, changes in the cell cycle, the ability to avoid apoptosis and the interaction between SDF-1 and CXCR4 expressed at the surface of AML cells.

## 2. Results

### 2.1. P1 and P2 Receptor mRNA Are Present in THP-1 Cells

**Figure 1:**
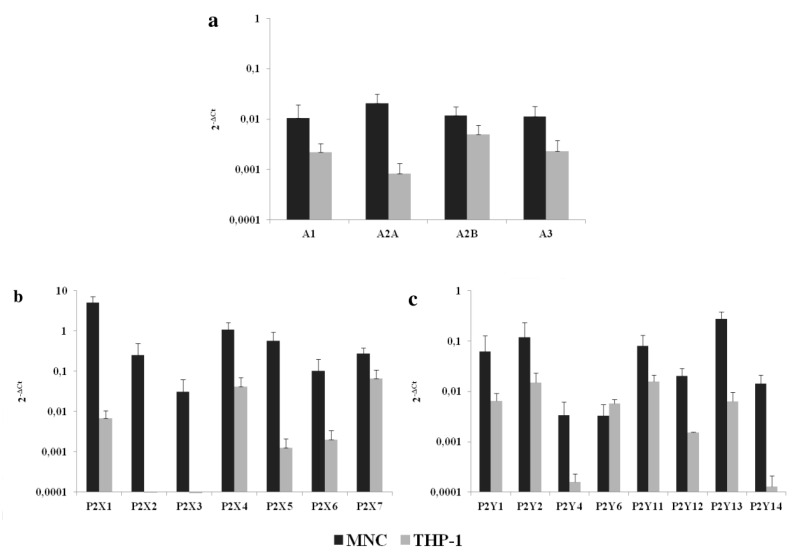
The mRNA expression of (**a**) P1, (**b**) P2X and (**c**) P2Y receptors in mononuclear cells (MNCs) and THP-1 cells. Data are presented as the mean ± SD.

The mRNA expressions of 17 human P1 and P2 receptor subtypes were evaluated. As shown in Figure 1, THP-1 cells expressed all P1 and P2Y receptor subtypes and some P2X receptors (P2X_1_, P2X_4_, P2X_5_, P2X_6_ and P2X_7_). P2X_2_ and P2X_3_ receptor mRNAs were not detected.

### 2.2. Adenine Nucleotides and Adenosine Inhibit the Growth of THP-1 Cells by Induction of Apoptosis and Cell Cycle Arrest

**Figure 2:**
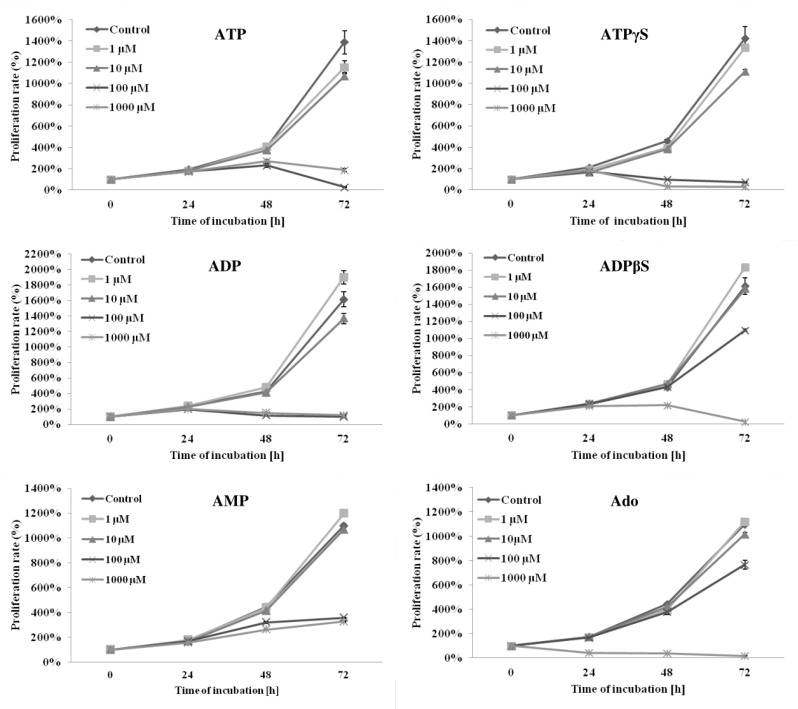
The effects of high (100–1000 μM), intermediate (10 μM) and low (1 μM) concentrations of adenine nucleotides or adenosine (Ado) on the proliferation of THP-1 cells. The proliferation rate (%) was evaluated after 24, 48 and 72 h of incubation by counting the number of cells using a flow cytometer. Data are presented as the mean ± SD of three different experiments. *p* < 0.05 compared with the unstimulated control cell culture.

We studied the antiproliferative and proapoptotic activities of adenine nucleotides and adenosine. At high concentrations (100–1000 μM), the natural adenine nucleotides (ATP, ADP and AMP), their nonhydrolyzable analogues (ATPγS and ADPβS) and adenosine significantly inhibited the proliferation of THP-1 cells (*p* < 0.05). At an intermediate concentration (10 μM), only some compounds (ATP, ATPγS ADP and adenosine) had significant inhibitory effects (*p* < 0.05). At a low concentration (1 μM), only ATP weakly inhibited proliferation, and, interestingly, stimulation of cell proliferation by ADP, ADPβS and AMP was observed (*p* < 0.05). The inhibitory effect of the studied compounds increased with time and was significantly more potent after 72 h of incubation compared to 24 or 48 h. In general, the inhibition potency of cell proliferation after 72 h of incubation with adenine nucleotides or adenosine increased with increasing concentration. Surprisingly, the exceptions were ATP and ADP, which inhibited proliferation significantly more at a concentration of 100 μM than 1000 μM (*p* < 0.05). This was not observed for their nonhydrolyzable analogues. At a concentration of 100 μM, the inhibition potencies (calculated as the percentage of the control) of ATP vs. ATPγS and ADP vs. ADPβS were as follows: ATP (2.0 ± 0.4%) > ATPγS (5.1 ± 0.6%) and ADP (6.1 ± 0.2%) > ADPβS (68.2 ± 3.8%) (*p* < 0.05). At 1000 μM, the trend was the opposite, and the inhibition potencies were the following: ATPγS (2.1 ± 0.1%) > ATP (13.6 ± 2.0%) and ADPβS (1.6 ± 0.2%) > ADP (7.4 ± 0.1%) (*p* < 0.05). The effects of adenine nucleotides and adenosine on THP-1 cell growth are shown in Figure 2.

**Figure 3:**
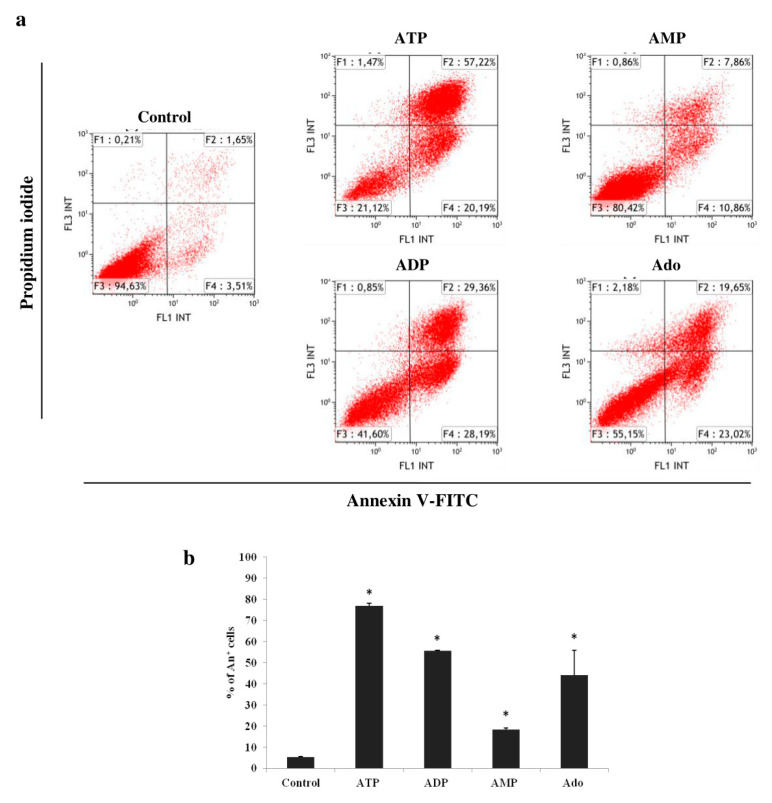
Effects of adenine nucleotides and adenosine (Ado) on apoptosis in THP-1 cells. (**a**) Representative flow cytometric analysis of THP-1 cells stained with annexin V-FITC and propidium iodide after 72 h of incubation with 1000 μM of adenine nucleotides or adenosine. (**b**) Percentage of apoptotic cells (Annexin V^+^, An^+^), induced by ATP, ADP, AMP and adenosine. Data are presented as the mean ± SD of three different experiments. * *p* < 0.05 compared with the unstimulated control cell culture.

The changes in the cell number presented by the proliferation rate are the result of cell division and death. Therefore, the effects of high concentrations (100–1000 μM) of ATP, ADP, AMP and adenosine on apoptosis and cell cycle were then assessed. The reduction in the cell number in the culture with 1000 μM of adenine nucleotides or adenosine was largely the result of the induction of apoptosis (Figure 3). All induced a significant increase in the percentage of apoptotic cells (Annexin V^+^), compared to the control, in the following order of potency: ATP > ADP = Ado > AMP (*p* < 0.05).

**Figure 4:**
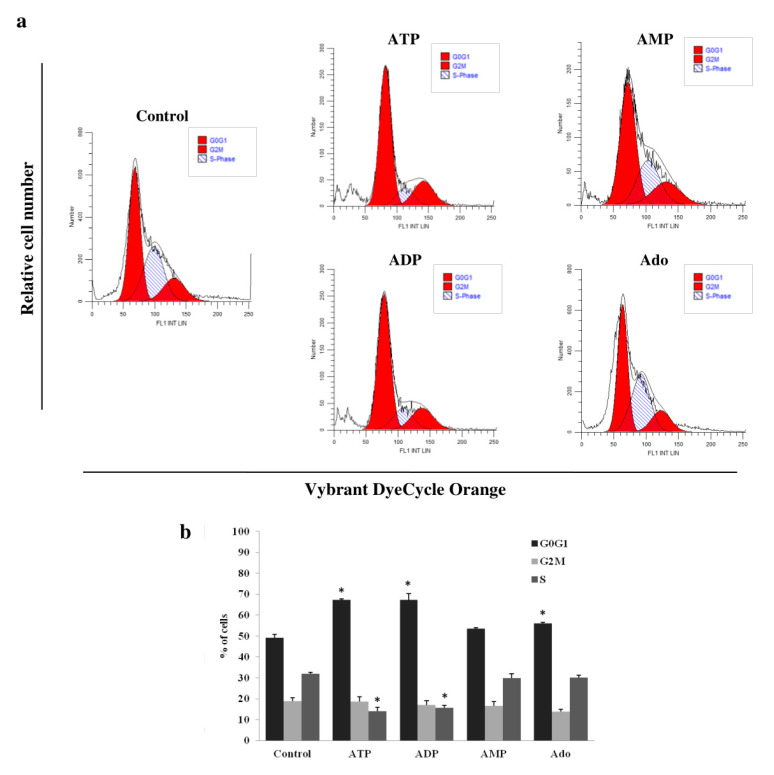
Effects of adenine nucleotides and adenosine (Ado) on the THP-1 cell cycle. (**a**) Representative flow cytometric analysis of THP-1 cells stained with Vybrant DyeCycle Orange after 72 h of incubation with 100 μM of adenine nucleotides or adenosine. (**b**) Changes in the percentage of cells in particular phases of the cell cycle (G_0_G_1_, S, and G_2_M) affected by ATP and ADP. Data are presented as the mean ± SD of three different experiments. * *p* < 0.05 compared to the unstimulated control cell culture.

Due to the high percentage of apoptotic cells after incubation with the tested purine compounds at a concentration of 1000 μM, we studied their effect on the cell cycle at a concentration of 100 μM. Among them, only ATP and ADP significantly changed the cell cycle compared to the control (Figure 4). They arrested the cell cycle in the G_0_G_1_ phase. After culturing THP-1 cells with ATP or ADP, the percentage of cells in the G_0_G_1_ phase increased (ATP, 67.2 ± 0.6% vs. control, 49.1 ± 1.6%; ADP, 67.3 ± 3.1% vs. control, 49.1 ± 1.6%; *p* < 0.05), while in the S phase, they decreased (ATP, 14.1 ± 1.7% vs. control, 32.0 ± 0.6%; ADP, 15.6 ± 1.2% vs. control, 32.0 ± 0.6%; *p* < 0.05). However, the percentage of cells in the G_2_M phase did not undergo statistically significant changes. The effects of both adenine nucleotides did not differ significantly.

### 2.3. Adenine Nucleotides and Adenosine Inhibit THP-1 Cell Chemotaxis towards SDF-1

**Figure 5:**
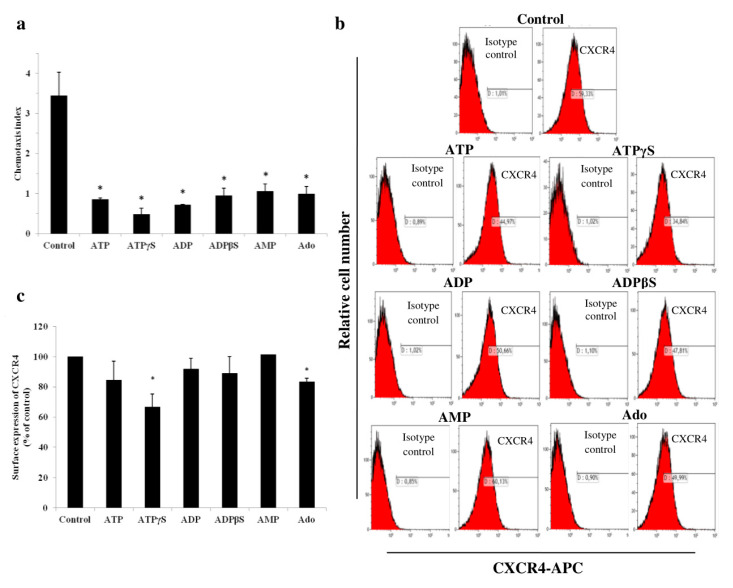
Adenine nucleotides and adenosine (Ado) modulate the stromal cell-derived factor-1/chemokine receptor type 4 (SDF-1/CXCR4) axis in THP-1 cells. (**a**) Chemotaxis of THP-1 cells towards SDF-1 was inhibited after 24 h of preincubation with 10 μM of adenine nucleotides or adenosine. The results are presented as the chemotaxis index. (**b**) Representative flow cytometric analysis of THP-1 cells stained using the APC-labelled mouse anti-CXCR4 antibody and the isotype control antibody after 24 h of incubation with 10 μM of adenine nucleotides or adenosine. (**c**) Effects of 10 μM of adenine nucleotides or adenosine on the surface expression of the CXCR4 receptor in THP-1 cells after 24 h of incubation. Data are presented as the mean ± SD of three different experiments. * *p* < 0.05 compared to the unstimulated control cell culture.

Then, we evaluated the effects of 10 μM adenine nucleotides and adenosine on the migration of THP-1 cells towards SDF-1. The preincubation time and concentration of adenine nucleotides and adenosine were chosen based on the proliferation assay to exclude the significant cytotoxic effect. All studied compounds inhibited cell chemotaxis compared to the control cell culture with comparable potency, corresponding to spontaneous migration (*p* < 0.05) (Figure 5a). We hypothesized that one of the reasons for this was a decrease in the cell surface expression of CXCR4. However, only ATPγS and adenosine significantly reduced the surface expression of CXCR4 compared to the control cell culture, i.e., by 33.2% and 16.5% of the control, respectively (Figure 5b,c).

## 3. Discussion

The role of purinergic signaling in AML is relatively poorly understood. Therefore, in this study, we investigated a range of actions, not only of the frequently evaluated ATP, but also of other extracellular adenine nucleotides and adenosine, on the leukemic THP-1 cell biology. We chose concentrations of adenine nucleotides and adenosine ranging from the physiological levels to higher concentrations observed in a leukemic microenvironment (1–1000 μM) [12]. Our research shows that ADP, AMP and adenosine at 100–1000 μM concentration are promising compounds with antiproliferative and cytotoxic effects, similar to ATP. Moreover, we showed that at 10 μM concentration, they inhibit cell migration towards SDF-1. Considering that these purinergic signaling molecules differ significantly in terms of activated receptors, the comparison of their antileukemic effects may be helpful in researching mechanisms of action and new drug targets.

We showed that THP-1 cells express a wide panel of P1 and P2 receptors. What is important is that these include receptors not only for ATP and adenosine, but also for the other extracellular adenine nucleotides, ADP and AMP. These receptors are involved in the modulation of functions in both normal [10,14,16] and leukemic bone marrow cells [17]. Among P2X receptors, P2X_1_, P2X_4_ and P2X_7_ showed the highest expression, with no or low P2X_2_, P2X_3_ and P2X_6_ expression, in agreement with the results of other authors [21,22]. A particularly interesting receptor is P2X_7_, which is activated only in the presence of high ATP concentrations [25]. Salvestrini et al. [17,22] compared the effects of high concentrations of ATP on AML cells and normal HSC or normal CD34^+^ cells purified from the peripheral blood, and demonstrated specific cytotoxicity to AML cells. They showed that ATP in the highest concentration we used, i.e., 1000 μM, reduces viability and induces apoptosis of AML cells, whereas normal cells were almost entirely unaffected, i.e., the growth decrease or induction of apoptosis were not observed. In the case of apoptosis, this was probably due to the different isoforms of the P2X_7_ receptor [17]. The ATP toxicity to AML cells described by Salvestrini et al. [17,22] was similar to what we observed on THP-1 cells. These features make ATP a promising candidate for developing therapeutic strategies with low toxicity to HSCs and antileukemic activity [17]. However, much less is known about the actions of other adenine nucleotides and the mechanisms based on purinergic receptor activation.

There is an imbalance in the processes of cell division and death in AML cells. Uncontrolled proliferation of AML cells is one of the main reasons for the progression of this group of malignancies. We found antiproliferative properties of high concentrations of adenine nucleotides and adenosine. Many authors have shown antineoplastic activity of ATP [26]. The cytotoxic effect of ATP on leukemic cells has been confirmed in several studies in AML cell lines and primary AML cells [17,22,27,28,29]. Cytotoxic activity was achieved in the range of 20 μM to 5 mM, depending on the cell model, the parameters of the experiments, and the laboratory techniques used. Interestingly, in our study 100 μM of ATP and ADP inhibited proliferation significantly more than 1000 μM, but it was not observed for their nonhydrolyzable analogues, ATPγS and ADPβS. We suppose that the reason for this phenomenon is the generation of larger amounts of adenosine at the lower concentration. Adenosine has been found to both inhibit and stimulate the proliferation of cancer cells [26]. As in our study, an overwhelming number of publications describe its antiproliferative activity, including in prostate cancer [30], ovarian cancer [31], cholangiocarcinoma [32] and AML [28,29]. ATP and ADP (in the low micromolar range) are competitive inhibitors of 5′-nucleotidase that hydrolyze AMP to adenosine and inorganic phosphate. Therefore, the degree of 5′-nucleotidase inhibition is much stronger at higher concentrations of ATP or ADP, which prevents the generation of adenosine [33]. However, the observed antiproliferative activities of 100–1000 μM of nonhydrolyzable analogues of ATP and ADP indicate that these compounds show their action through P2 receptor activation, not just adenosine generation. This issue was considered by Seetulsingh-Goorah et al. [27] and Conigrave et al. [28], who showed that the mechanism of inhibition of HL-60 cell growth by ATP is dependent on adenosine generation and its extracellular accumulation. The first study also indicated the participation of P2 receptors for ATP at concentrations below 250 μM. Among the P2 receptors, P2X_5_, P2X_7_, P2Y_1_, P2Y_2_ and P2Y_11_ contribute to the inhibition of cancer cell growth by adenine nucleotides [26]. In addition, P2X_1_, P2X_4_ and P2X_5_ receptors are noteworthy because increased expression has been demonstrated on the surface of pediatric primary AML cells, as previously mentioned [21]. The role of the P2X_7_ receptor in ATP-mediated inhibition of AML cell proliferation is best known among purinergic receptors [22,34]. However, it is highly likely that ATP antileukemic actions are dependent on the activation of more than one receptor. However, explaining receptor-dependent mechanisms is a major challenge considering the large number of purinergic receptors and the need for numerous experiments using selective antagonists.

The inhibitory effect of AMP on cell proliferation was the weakest. Considering the antiproliferative effect of extracellular nucleotides, we find that AMP is the least known nucleotide. This is due to the indirect effects of AMP described in the literature (by generating adenosine), a lack of identified receptors for AMP and the unavailability of its nonhydrolyzable analogues (AMPαS is currently available). The ability of AMP to inhibit proliferation by hydrolysis to adenosine has been described in HL-60 cells [27,28]. We cannot exclude this mechanism in our study, but the fact that 100 μM of AMP is more potent than 100 μM of adenosine suggests receptor involvement. The potential receptors for AMP are A_1_ [35] and A_2B_ [36].

Interestingly, 1 μM of ADP and ADPβS stimulated proliferation of THP-1 cells. As the concentration increased, the described effect disappeared (10 μM), and then changed into inhibition (100–1000 μM). There have been no reports of the stimulating effect of low concentrations of ADP on cancer cells. However, a similar effect was observed in breast cancer [37] and ovarian cancer [38] cells in response to low micromolar ATPγS concentrations. The stimulating effect of ADP and ADPβS may result from the activation of the P2Y_12_ or P2Y_13_ receptor, but not P2Y_1_, because a concentration of 1 μM is insufficient to stimulate in the latter case [39].

Next, we analyzed the effects of high concentrations of adenine nucleotides and adenosine on apoptosis and the cell cycle. Studies carried out over the past 30 years have led to the conclusion that ATP and its derivatives have a strong cytotoxic effect on various types of cancer cells [26]. We assayed the induction of apoptosis after 72 h of incubation with 1000 μM of the evaluated compounds. These culture conditions resulted in the strongest cytotoxic effect. ATP was the most potent, inducing apoptosis in over 75% of cells. There have been several studies confirming the proapoptotic activity of ATP on AML cells. The effects of incubation of cells with high ATP concentrations were the induction of selected proapoptotic genes [22], the activation of caspases [17,29] and the transfer of phosphatidylserine to the outside of the cell membrane [17,22,29,34]. Factors significantly affecting the level of apoptosis were ATP concentration in the culture medium and incubation time (an increase with time). Yoon et al. [29] found that the cells incubated with 10–100 μM of ATP did not undergo significant apoptosis. However, several studies emphasized that significant ATP activity only appeared at a concentration of 1000 μM and intensified with its increase [17,22,29,34]. The authors also indicated the involvement of the P2X_7_ receptor in the mechanism of induction of AML cell apoptosis by ATP [17,29,34]. The property that distinguishes the P2X_7_ receptor from other ATP receptors is the high concentration of nucleotide necessary for its activation (100–1000 μM) [25]. Therefore, among the different types of leukemia cell lines, there are those that do not express this receptor and do not respond to ATP by induction of apoptosis [29,34]. The effect of ADP and AMP on cancer cell apoptosis has not yet been established. Our results showed that ADP, second in terms of potency, induced apoptosis in more than half of THP-1 cells. Adenosine is slightly less potent. Due to the potency of the proapoptotic activity, ADP and adenosine are no less promising compounds than ATP. An ATP and ADP concentration of 100 μM caused an increase in the percentage of THP-1 cells in the G_0_G_1_ phase, with a simultaneous decrease in the percentage of cells in the S phase, which indicates cell cycle arrest in the G_0_G_1_ phase. We present the effect of a 100 μM concentration due to the high percentage of apoptotic cells after incubation with the higher 1000 μM concentration. This was confirmed by the results of several studies, which were discussed in more detail in the section on apoptosis [17,22,29,34]. The first reports about the effects of ATP and its degradation products on the cancer cell cycle appeared in 1983 [40]. They described cell cycle arrest in the S phase in response to ATP and ADP, and the direction of cells to the apoptosis pathway. Until now, only the effect of ATP on the AML cell cycle has been evaluated, confirming its involvement in the negative regulation of the cell cycle. There is no information about the effect of ADP. Yoon et al. [29], who used the HL-60 and F-36P cell lines as an AML model, obtained a similar ATP effect to that observed in our study. But the effect only appeared at a concentration of 10 mM, which may be due to the much shorter incubation time (24 h). In the study by the other group, the results in primary AML cells showed an increase in the percentage of cells in the G_0_ phase, without significant changes in the G_1_ and S phases. This was accompanied by the induction of a cell cycle inhibitor gene and the repression of a cell cycle regulatory protein gene such as cyclins, cyclin-dependent protein kinases, and transcription factors associated with proliferation [22]. Adenosine caused only a slight increase in the percentage of THP-1 cells in the G_0_G_1_ phase. However, Lee et al. [41] showed that the incubation of HL-60 cells with 25 μM of thio-Cl-IB-MECA, a synthetic A_3_ receptor agonist, led to changes in the expression of cell cycle regulatory proteins, a decrease in the expression of cyclin D1 (responsible for the transition from G_1_ to S) and c-myc oncogen, and cell retention in the G_0_/G_1_ phase. A higher concentration of 50 μM caused cell apoptosis. The weak effect of adenosine compared to that described after the activation of adenosine receptors by other authors is most likely due to the use of synthetic agonists with different pharmacological properties and different cell models. The THP-1 cell cycle arrest in the G_0_/G_1_ phase observed by our group proves the cytostatic effect of ATP and ADP. Arresting the cell cycle at this stage may have the benefit of reducing the proliferation of leukemia cells and directing them to the apoptosis pathway [42].

At this point, it is worth summarizing that the effect of the tested compounds on the growth and survival of THP-1 cells is not the same, both in terms of potency and range of activities. ATP is characterized by the highest potency and widest range of effects, and is responsible for the cell cycle arrest and the apoptosis induction. Compared to ATP, the effect of ADP is slightly weaker. Adenosine mostly has a cytotoxic effect with induction of apoptosis. In contrast, AMP demonstrated only a weak cytotoxic effect without affecting the cell cycle. These differences are probably due to a different panel of receptors being responsible for mediating their effects. This was noted in the discussion on the results. Hydrolysis by membrane ectonucleotidases may also play a role, although we limited its effect by using nonhydrolyzable analogues in our experiments.

The effect of adenine nucleotides and adenosine on SDF-1-dependent migration was also interesting. SDF-1 and its CXCR4 receptor, whose expression is often increased in AML cells, play an important role in the pathogenesis and recurrence of leukemia. The SDF-1/CXCR4 axis is currently one of the more promising targets for AML treatment [43]. To the best of our knowledge, this is the first study simultaneously investigating the effects of adenine nucleotides and adenosine on AML cell migration towards SDF-1. We showed inhibition of THP-1 cell migration towards SDF-1 by these signaling molecules. The antimigratory action of ATP and UTP in AML cells was studied by Salvestrini et al. [22]. They indicated that UTP, but not ATP, inhibited the migration of primary AML cells towards SDF-1 by P2Y_2_ and P2Y_4_ receptors. Both nucleotides reduced the homing and engraftment capacity of LSCs and AML blasts to immunodeficient mice bone marrow. One of the reasons for the inhibition of SDF-1 migration we observed is the partial reduction in surface expression of the CXCR4 receptor by ATPγS and adenosine. The surface density of CXCR4 is positively correlated with SDF-1-induced migration [43]. Other authors have indicated the ability of adenosine to upregulate the CXCR4 receptor in carcinoma cells [44] or tumor stromal cells [45]. However, Salvestrini et al. [22] stated that neither modulation of the expression of the cell membrane nor intracytoplasmic CXCR4 cause the inhibition of AML cell migration by UTP. A partial decrease or no decrease in expression suggests that other mechanisms are also involved in the inhibition of THP-1 cell chemotaxis. The described antimigratory activity may be associated with disruption of receptor function and cytoskeleton remodeling. Optimal function of CXCR4 depends on its relocation from the intracellular stores and incorporation into membrane lipid rafts. Changes in the composition of lipid rafts or disturbances in the recruitment of CXCR4 to lipid rafts will result in the inhibition of cell migration towards SDF-1. Therefore, the modulation of lipid rafts could provide a new antimigratory strategy [46,47]. Another reason might be disturbances of cytoskeleton remodeling. SDF-1-dependent AML cell migration and adhesion are closely associated with the reorganization of F-actin microfilaments [48]. These changes were observed for endothelial cells from human breast carcinoma, but not for normal human microvascular endothelial cells, after incubation with ATP and ADP (>20 μM). Avanzato et al. [49] indicated the involvement of P2X_7_ and P2Y_11_ receptors in cytoskeleton remodeling. Changes in the SDF-1/CXCR4 axis may contribute to the weakening of the interaction of leukemia cells with the bone marrow stroma, responsible for the presence of chemoresistance, MRD, and an increase in the risk of recurrence. Disease relapse remains the highest cause of mortality after HSC transplantation.

Our study has some limitations. Natural adenine nucleotides added at the beginning of the experiment get gradually degraded. Over the course of the experiment, cells were exposed to mixed concentrations of further degradation products of adenine nucleotides. This makes the results more difficult to interpret. On the other hand, ATP degradation is a normally occurring process in the bone marrow niche. To limit the impact of degradation products, nonhydrolyzable ATP and ADP analogues were included. Therefore, an important issue is to consider the advantages and disadvantages of natural nucleotides or their synthetic counterparts with a specific range of action as potential purinergic-based drugs.

## 4. Materials and Methods

### 4.1. Reagents

ATP, ADP, AMP, adenosine and the nonhydrolyzable ATP and ADP analogues ATPγS and ADPβS were obtained from Sigma-Aldrich (St. Louis, MO, USA). SDF-1 was obtained from PeproTech (London, UK).

### 4.2. Cell Culture

The study was conducted on AML cell line THP-1, obtained from ATCC. Cells were cultured in RPMI-1640 medium (Sigma-Aldrich, St. Louis, MO, USA) supplemented with 10% fetal bovine serum (FBS) (Gibco, Brazil), penicillin (100 IU/mL) and streptomycin (10 μg/mL) (Sigma-Aldrich). The cell culture was maintained in a humidified atmosphere at 37 °C in 5% CO_2_. The medium was changed every 2–3 days.

### 4.3. Real-Time PCR

Total RNA was isolated using the Rneasy Mini Kit (QIAGEN, Valencia, CA, USA) following the manufacturer’s instructions. Reverse transcription was performed using the First Strand cDNA Synthesis Kit (Thermo Scientific, Waltham, MA, USA) on a T100 thermal cycler (BIO-RAD, Philadelphia, PA, USA) according to the manufacturer’s instructions. The quantitative assessment of mRNA levels was performed by the real-time PCR (qRT-PCR) technique on an ABI 7500 Fast instrument (Applied Biosystems, Foster City, CA, USA) with Power SYBR Green PCR Master Mix reagent (Applied Biosystems). The qRT-PCR parameters were as follows: 95 °C for 15 s, 40 cycles of 95 °C for 15 s and 60 °C for 1 min. The sequences of primers used for quantitative real time PCR analysis are shown in Table 1. The reaction characteristics and the primers used in the study were designed to achieve equal or similar reaction efficiencies. The obtained amplicons were in a narrow length range, i.e., 70–150 bp. These small amplicons were favored because they promote high-efficiency assays. Every reaction produced melting curve data showing the presence of only a single product and an appropriate amplification curve. Primers were paired with very similar melting temperatures and G/C content. This allowed us to estimate reaction efficiency to fit into a 90% < E < 110% range. qRT-PCR data were analyzed using the ΔCt method. Values were normalized to β2-microglobulin, and were expressed as relative expression levels.

### 4.4. Proliferation

The cells were seeded into 24-well plates at an initial density of 0.025 × 106 cells/mL in complete medium. They were cultured in the presence of adenine nucleotides or adenosine in the concentration range of 1–1000 μM, or without the addition of these compounds (control). The initial number of cells was counted prior to incubation. Then, the number of cells was counted 24, 48 and 72 h after the start of the culture. At these time points, the cells were collected and counted using a flow cytometer (Navios, Beckman Coulter). The results are presented as the proliferation rate (%), calculated as follows: (number of cells after incubation/initial number of cells before incubation) × 100%.

### 4.5. Apoptosis

Apoptosis was measured with an Annexin V-FITC Apoptosis Kit (BD Bioscience, San Diego, CA, USA). The cells were seeded into 12-well plates at an initial density of 0.150 × 106 cells/mL in complete medium. After 72 h of incubation with or without 1000 μM of adenine nucleotides or adenosine, 1 × 106 cells were washed with phosphate buffer saline (PBS) (Corning, Manassas, VA, USA), resuspended in 100 μL of buffer and stained with FITC-conjugated Annexin-V and propidium iodide. The cells were stained according to the manufacturer’s instructions and analyzed by flow cytometry. The histograms were analyzed with the Kaluza analysis software (v2.1, Beckman Culture, Brea, CA, USA). The results are expressed as the percentage of apoptotic cells (annexin V^+^ cells).

### 4.6. Cell Cycle Analysis

The cell cycle was assayed with Vybrant DyeCycle Orange Stain (Invitrogen, Thermo Fisher Scientific, Eugene, OR, USA). The cells were seeded into 12-well plates at an initial density of 0.150 × 106 cells/mL in complete medium. After 72 h of incubation with or without 100 μM of adenine nucleotides or adenosine, the cells were washed with PBS and resuspended in 1 mL of RPMI 1640 medium with 10% FBS at a density of 106 cells/mL. The cells were stained according to the manufacturer’s instructions and analyzed by flow cytometry. The histograms were analyzed with the ModFit LT 4.1 software (Verity Software House, Topsham, ME, USA). The results are presented as the percentage of cells in particular phases of the cycle (G_0_G_1_, S, and G_2_M).

### 4.7. Chemotaxis Assay

Chemotaxis of THP-1 cells towards SDF-1 was tested using 8-μm pore size Transwell inserts (Transwell, Costar, Corning). The cells were seeded into 24-well plates at a density of 0.300 × 106 cells/mL in RPMI-1640 medium with 0.5% BSA and incubated for 24 h with or without 10 μM of adenine nucleotides or adenosine. Then, the cells were washed with PBS, resuspended in 120 μL of RPMI-1640 medium with 0.5% BSA and placed in the upper chambers. RPMI-1640 medium (650 μL), with or without SDF-1 (150 ng/mL), was added to the bottom chambers. After 6 h of incubation in a humidified atmosphere at 37 °C in 5% CO_2_, the inserts were removed, and the cells that were transmigrated into the lower chamber were counted. The number of migrating cells was counted with flow cytometry. The results are shown as the chemotaxis index, calculated as follows: number of cells that migrated to medium with SDF-1 after preincubation with or without (control) tested compounds/number of cells that migrated to medium without SDF-1.

### 4.8. CXCR4 Receptor Expression Analysis by Flow Cytometry

The surface expression of the CXCR4 receptor was analyzed using the APC-labelled mouse antihuman CXCR4 antibody (BD Bioscience). The cells were incubated with or without 10 μM of adenine nucleotides or adenosine, as in the chemotaxis assay procedure. Subsequently, the cells were washed and resuspended in 100 μL of PBS and incubated with 10 μL of conjugated antibody or its isotype control for 30 min at 4 °C (in the dark). After staining, the cells were washed, resuspended in 400 μL of PBS and analyzed by flow cytometry. The results are presented as the percentage relative to the control cells cultured in the medium without the tested compounds.

### 4.9. Statistical Analysis

The results are expressed as means ± standard deviation (SD) of three different experiments. Statistical comparisons were performed using the t test. *p* < 0.05 was considered statistically significant.

## 5. Conclusions

In conclusion, not only ATP, but also other adenine nucleotides and adenosine have several antileukemic actions. High micromolar concentrations (100–1000 μM) of extracellular ATP, ADP, AMP and adenosine inhibit the growth of THP-1 leukemia cells by arresting the cell cycle and/or inducing apoptosis. Among them, ATP has the most potent antileukemic effect, expressed both as cell cycle arrest and apoptosis induction. A similar effect, although slightly weaker, was observed for ADP. Adenosine was characterized by significant cytotoxicity by the induction of apoptosis, but weakly affected the cell cycle. The least potent of the studied molecules proved to be AMP, which exerted a cytotoxic effect without affecting the cell cycle. The differences in the response to these compounds come from a different panel of receptors through which they mediate their effects, although it is possible that a naturally occurring process of hydrolysis by ectonucleotidases plays a role. A very surprising observation was the inhibition of THP-1 cell migration towards SDF-1 by low micromolar concentrations (10 μM) of adenine nucleotides and adenosine. One of the mechanisms of action of ATPγS and adenosine was a reduction in CXCR4 surface expression, but there must be others. These observations indicate that not only ATP, but also its derivatives, especially ADP and adenosine, are promising compounds in the search for new antileukemic drugs. The properties of adenine nucleotides and adenosine could be used to develop promising novel agents that act directly on the growth and survival of AML cells, as well as their interactions with the bone marrow stroma. Therefore, more research is needed to gain a better understanding of the receptor-dependent mechanisms of the antileukemic action of purinergic signaling molecules.

## Figures and Tables

**Table 1:** Sequences of primers used for quantitative real time PCR analysis.

Gene	Forward	Reverse
*B2M*	5′-AATGCGGCATCTTCAAACCT-3′	5′-TGACTTTGTCACAGCCCAAGATA-3′
*ADORA1*	5′-TGCGAGTTCGAGAAGGTCATC-3′	5′-GAGCTGCTTGCGGATTAGGTA-3′
*ADORA2A*	5′-CGAGGGCTAAGGGCATCATTG-3′	5′-CTCCTTTGGCTGACCGCAGTT-3′
*ADORA2B*	5′-CTCTTCCTCGCCTGCTTCGTG-3′	5′-TTATACCTGAGCGGGACACAG-3′
*ADORA3*	5′-TACATCATTCGGAACAAACTC-3′	5′-GTCTTGAACTCCCGTCCATAA-3′
*P2RX1*	5′-CGCCTTCCTCTTCGAGTATGA-3′	5′AGATAACGCCCACCTTCTTATTACG-3′
*P2RX2*	5′-GCCTACGGGATCCGCATT-3′	5′-TGGTGGGAATCAGGCTGAAC-3′
*P2RX3*	5′-GCTGGACCATCGGGATCA-3′	5′-GAAAACCCACCCTACAAAGTAGGA-3′
*P2RX4*	5′-CCTCTGCTTGCCCAGGTACTC-3′	5′-CCAGGAGATACGTTGTGCTCAA-3′
*P2RX5*	5′-CTGCCTGTCGCTGTTCGA-3′	5′-GCAGGCCCACCTTCTTGTT-3′
*P2RX6*	5′-AGGCCAGTGTGTGGTGTTCA-3′	5′-TCTCCACGGGGCACCAACTC-3′
*P2RX7*	5′-TCTTCGTGATGACAAACTTTCTCAA-3′	5′-GTCCTGCGGGTGGGATACT-3′
*P2RY1*	5′-CGTGCTGGTGTGGCTCATT-3′	5′-GGACCCCGGTACCTGAGTAGA-3′
*P2RY2*	5′-CCAGGTCCAGGCGTGTGCAT-3′	5′-CATCAGGGTTGTGGCCGGAGC-3′
*P2RY4*	5′-TCGCCTCGCAGGCCTTCTCT-3′	5′-CAGGCAGGGCACGCCAAAGA-3′
*P2RY6*	5′-GGTGCGGTCCTCAGTGAGCC-3′	5′-CGCCAGCACCGCCGAATACA-3′
*P2RY11*	5′-GGCTGAGGATCGGCACGGGA-3′	5′-ATGGGCCACAGGAAGTCCCCC-3′
*P2RY12*	5′-AGGTCCTCTTCCCACTGCTCTA-3′	5′-CATCGCCAGGCCATTTGT-3′
*P2RY13*	5′-GAGACACTCGGATAGTACAGCTGGTA-3′	5′-GCAGGATGCCGGTCAAGA-3′
*P2RY14*	5′-TTCCTTTCAAGATCCTTGGTGACT-3′	5′-ACGGAGACCCTGCACACAAA-3′

## Abbreviations

AML	acute myeloid leukemia
CD73	ecto-5′-nucleotidase
CXCR4	chemokine receptor type 4
E-NTPDase1/CD39	ecto-nucleoside triphosphate diphosphohydrolase 1
HSC	hematopoietic stem cells
MRD	minimal residual disease
LSC	leukemic stem cells
SDF-1	stromal cell-derived factor-1

## References

[1] Saultz J.N., Garzon R. (2016). Acute Myeloid Leukemia: A Concise Review. J. Clin. Med..

[2] World Health Organization Review of Cancer Medicines on the WHO List of Essential Medicines. http://www.who.int/selection_medicines/committees/expert/20/applications/AML_APL.pdf.

[3] National Cancer Institute Cancer Stat Facts: Leukemia—Acute Myeloid Leukemia (AML). https://seer.cancer.gov/statfacts/html/amyl.html.

[4] Fey M.F., Buske C., ESMO Guidelines Working Group (2013). Acute myeloblastic leukaemias in adult patients: ESMO Clinical Practice Guidelines for diagnosis, treatment and follow-up. Ann. Oncol..

[5] Fiegl M., Hiddemann W. (2016). Epidemiology, pathogenesis, and etiology of acute leukemia. Handbook of Acute Leukemia.

[6] Prada-Arismendy J., Arroyave J.C., Röthlisberger S. (2017). Molecular biomarkers in acute myeloid leukemia. Blood Rev..

[7] Peled A., Tavor S. (2013). Role of CXCR4 in the pathogenesis of acute myeloid leukemia. Theranostics.

[8] Duarte D., Hawkins E.D., Lo Celso C. (2008). The interplay of leukemia cells and the bone marrow microenvironment. Blood.

[9] Kumar R., Godavarthy P.S., Krause D.S. (2018). The bone marrow microenvironment in health and disease at a glance. J. Cell Sci..

[10] Rossi L., Salvestrini V., Ferrari D., Di Virgilio F., Lemoli R.M. (2012). The sixth sense: Hematopoietic stem cells detect danger through purinergic signaling. Blood.

[11] Di Virgilio F., Adinolfi E. (2017). Extracellular purines, purinergic receptors and tumor growth. Oncogene.

[12] Pellegatti P., Raffaghello L., Bianchi G., Piccardi F., Pistoia V., Di Virgilio F. (2008). Increased level of extracellular ATP at tumor sites: In vivo imaging with plasma membrane luciferase. PLoS ONE.

[13] Di Virgilio F. (2012). Purines, purinergic receptors, and cancer. Cancer Res..

[14] Ratajczak M.Z., Adamiak M., Plonka M., Abdel-Latif A., Ratajczak J. (2018). Mobilization of hematopoietic stem cells as a result of innate immunity-mediated sterile inflammation in the bone marrow microenvironment-the involvement of extracellular nucleotides and purinergic signaling. Leukemia.

[15] Filippin K.J., de Souza K.F.S., de Araujo Júnior R.T., Torquato H.F.V., Dias D.A., Parisotto E.B., Ferreira A.T., Paredes-Gamero E.J. (2020). Involvement of P2 receptors in hematopoiesis and hematopoietic disorders, and as pharmacological targets. Purinergic Signal..

[16] Adamiak M., Bujko K., Brzezniakiewicz-Janus K., Kucia M., Ratajczak J., Ratajczak M.Z. (2019). The inhibition of CD39 and CD73 cell surface ectonucleotidases by small molecular inhibitors enhances the mobilization of bone marrow residing stem cells by decreasing the extracellular level of adenosine. Stem Cell Rev. Rep..

[17] Salvestrini V., Orecchioni S., Talarico G., Reggiani F., Mazzetti C., Bertolini F., Orioli E., Adinolfi E., Di Virgilio F., Pezzi A. (2017). Extracellular ATP induces apoptosis through P2X7R activation in acute myeloid leukemia cells but not in normal hematopoietic stem cells. Oncotarget.

[18] Burnstock G. (2018). Purine and purinergic receptors. Brain Neurosci. Adv..

[19] Borea P.A., Gessi S., Merighi S., Vincenzi F., Varani K. (2018). Pharmacology of adenosine receptors: The state of the art. Physiol. Rev..

[20] Zhang X., Zheng G., Ma X., Yang Y., Li G., Rao Q., Nie K., Wu K. (2004). Expression of P2X7 in human hematopoietic cell lines and leukemia patients. Leuk. Res..

[21] Chong J.H., Zheng G.G., Zhu X.F., Guo Y., Wang L., Ma C.H., Liu S.Y., Xu L.L., Lin Y.M., Wu K.F. (2010). Abnormal expression of P2X family receptors in Chinese pediatric acute leukemias. Biochem. Biophys. Res. Commun..

[22] Salvestrini V., Zini R., Rossi L., Gulinelli S., Manfredini R., Bianchi E., Piacibello W., Caione L., Migliardi G., Ricciardi M.R. (2012). Purinergic signaling inhibits human acute myeloblastic leukemia cell proliferation, migration, and engraftment in immunodeficient mice. Blood.

[23] Dulphy N., Henry G., Hemon P., Khaznadar Z., Dombret H., Boissel N., Bensussan A., Toubert A. (2014). Contribution of CD39 to the immunosuppressive microenvironment of acute myeloid leukaemia at diagnosis. Br. J. Haematol..

[24] Lemoli R.M., Ferrari D., Fogli M., Rossi L., Pizzirani C., Forchap S., Chiozzi P., Vaselli D., Bertolini F., Foutz T. (2004). Extracellular nucleotides are potent stimulators of human hematopoietic stem cells in vitro and in vivo. Blood.

[25] Abbracchio M.P., Burnstock G., Verkhratsky A., Zimmerman H. (2009). Purinergic signalling in the nervous system: An overview. Trends Neurosci..

[26] Burnstock G., Di Virgilio F. (2013). Purinergic signalling and cancer. Purinergic Signal..

[27] Seetulsingh-Goorah S.P., Stewart B.W. (1998). Growth inhibition of HL-60 cells by extracellular ATP: Concentration-dependent involvement of a P2 receptor and adenosine generation. Biochem. Biophys. Res. Commun..

[28] Conigrave A.D., van der Weyden L., Holt L., Jiang L., Wilson P., Christopherson R.I., Morris M.B. (2000). Extracellular ATP-dependent suppression of proliferation and induction of differentiation of human HL-60 leukemia cells by distinct mechanisms. Biochem. Pharmacol..

[29] Yoon M.J., Lee H.J., Kim J.H., Kim D.K. (2006). Extracellular ATP induces apoptotic signaling in human monocyte leukemic cells, HL-60 and F-36P. Arch. Pharm. Res..

[30] Lertsuwan K., Peters W., Johnson L., Lertsuwan J., Marwa I., Sikes R.A. (2017). Purinergic receptor expression and cellular responses to purinergic agonists in human prostate cancer cells. Anticancer Res..

[31] Joshaghani H.R., Jafari S.M., Aghaei M., Panjehpour M., Abedi H. (2017). A3 adenosine receptor agonist induce G1 cell cycle arrest via Cyclin D and cyclin-dependent kinase 4 pathways in OVCAR-3 and Caov-4 cell lines. J. Cancer Res. Ther..

[32] Lertsuwan J., Ruchirawat M. (2017). Inhibitory effects of ATP and adenosine on cholangiocarcinoma cell proliferation and motility. Anticancer Res..

[33] Zimmermann H. (1992). 5′-Nucleotidase: Molecular structure and functional aspects. Biochem. J..

[34] Zhang X., Meng L., He B., Chen J., Liu P., Zhao J., Zhang Y., Li M., An D. (2009). The role of P2X7 receptor in ATP-mediated human leukemia cell death: Calcium influx-independent. Acta Biochim. Biophys. Sin. (Shanghai).

[35] Rittiner J.E., Korboukh I., Hull-Ryde E.A., Jin J., Janzen W.P., Frye S.V., Zylka M.J. (2012). AMP is an adenosine A1 receptor agonist. J. Biol. Chem..

[36] Holien J.K., Seibt B., Roberts V., Salvaris E., Parker M.W., Cowan P.J., Dwyer K.M. (2018). AMP and adenosine are both ligands for adenosine 2B receptor signaling. Bioorg. Med. Chem. Lett..

[37] Dixon C.J., Bowler W.B., Fleetwood P., Ginty A.F., Gallagher J.A., Carron J.A. (1997). Extracellular nucleotides stimulate proliferation in MCF-7 breast cancer cells via P2-purinoceptors. Br. J. Cancer.

[38] Batra S., Fadeel I. (1994). Release of intracellular calcium and stimulation of cell growth by ATP and histamine in human ovarian cancer cells (SKOV-3). Cancer Lett..

[39] Jacobson K.A. (2010). P2X and P2Y receptors. Tocris Sci. Rev. Ser..

[40] Rapaport E. (1983). Treatment of human tumor cells with ADP or ATP yields arrest of growth in the S phase of the cell cycle. J. Cell Physiol..

[41] Lee E.J., Min H.Y., Chung H.J., Park E.J., Shin D.H., Jeong L.S., Lee S.K. (2005). A novel adenosine analog, thio-Cl-IB-MECA, induces G_0_/G_1_ cell cycle arrest and apoptosis in human promyelocytic leukemia HL-60 cells. Biochem. Pharmacol..

[42] Schnerch D., Yalcintepe J., Schmidts A., Becker H., Follo M., Engelhardt M., Wäsch R. (2012). Cell cycle control in acute myeloid leukemia. Am. J. Cancer Res..

[43] Cho B.S., Kim H.J., Konopleva M. (2017). Targeting the CXCL12/CXCR4 axis in acute myeloid leukemia: From bench to bedside. Korean J. Intern. Med..

[44] Richard C.L., Tan E.Y., Blay J. (2006). Adenosine upregulates CXCR4 and enhances the proliferative and migratory responses of human carcinoma cells to CXCL12/SDF-1α. Int. J. Cancer.

[45] Sorrentino C., Miele L., Porta A., Pinto A., Morello S. (2016). Activation of the A2B adenosine receptor in B16 melanomas induces CXCL12 expression in FAP-positive tumor stromal cells, enhancing tumor progression. Oncotarget.

[46] Ratajczak M.Z., Adamiak M. (2015). Membrane lipid rafts, master regulators of hematopoietic stem cell retention in bone marrow and their trafficking. Leukemia.

[47] Tabe Y., Jin L., Iwabuchi K., Wang R.Y., Ichikawa N., Miida T., Cortes J., Andreeff M., Konopleva M. (2012). Role of stromal microenvironment in nonpharmacological resistance of CML to imatinib through Lyn/CXCR4 interactions in lipid rafts. Leukemia.

[48] Li X., Guo H., Yang Y., Meng J., Liu J., Wang C., Xu H. (2014). A designed peptide targeting CXCR4 displays anti-acute myelocytic leukemia activity in vitro and in vivo. Sci. Rep..

[49] Avanzato D., Genova T., Fiorio Pla A., Bernardini M., Bianco S., Bussolati B., Mancardi D., Giraudo E., Maione F., Cassoni P. (2016). Activation of P2X7 and P2Y11 purinergic receptors inhibits migration and normalizes tumor-derived endothelial cells via cAMP signaling. Sci. Rep..

